# Possibilities and limits in the treatment of congenital diaphragmatic hernia

**Published:** 2014-09-25

**Authors:** R Georgescu, L Chiuţu, R Nemeş, I Georgescu, A Stoica, E Georgescu

**Affiliations:** * 1st Surgical Clinic, Craiova University of Medicine and Pharmacy, Craiova, Romania; ** Intensive Care Unit, Craiova University of Medicine and Pharmacy, Craiova, Romania; *** Pediatric Surgical Clinic, Craiova University of Medicine and Pharmacy, Craiova, Romania

**Keywords:** congenital diaphragmatic hernias, malformations, diaphragm embryogenesis, conventional mechanical ventilation, prenatal diagnosis

## Abstract

Abstract

Aim: to establish a therapeutic strategy that will improve the prognosis and increase the survival rate in congenital diaphragmatic hernia.

Material and method: 14 congenital diaphragmatic hernias (incidence 1/1597 live births, 12 boys and 2 girls with a sex ratio of 6/1, 10 term infants and 4 preterm first degree, 11 natural births and 3 by caesarean section) admitted to the Clinic of Pediatric Surgery Craiova, in a 5-year period (2007-2012), were analyzed from the therapeutic point of view. The "tension free" primary suture was the main surgical procedure to repair the diaphragmatic defect in all cases, preceded by a period of preoperative resuscitation and stabilization (2.8 days on average).

Results: We registered a survival rate of 64.29% and a postoperative mortality rate of 35.71%.

Conclusions: delayed surgery preceded by a period of the preoperative respiratory resuscitation and stabilization (24-72 hours on average) significantly reduced postoperative mortality and increased the survival rate.

## Introduction

Congenital diaphragmatic hernias are among the most severe congenital malformations, their almost constant association with pulmonary hypoplasia and their concomitance with other malformations (cardiovascular, digestive, neurological, skeletal, etc.) making them responsible, until recently, for a very high mortality rate (70-80%), although the malformation can be surgically treated in most cases. Developing deeper knowledge of the diaphragm embryogenesis and a proper understanding of the consequences that the diaphragmatic hernia has upon the development and upon lung function, the prenatal diagnosis which is possible with the introduction of prenatal ultrasound imaging as a routine test in monitoring pregnancy, the development of a wider range of modern respiratory resuscitation methods (mechanical ventilation, surfactant, nitric oxide, ECMO) and the unanimous acceptance of the concept of delayed surgery preceded by a preoperative resuscitation and stabilization period, led to the improvement of prognosis and significantly increased the survival rate. 

## Material and method 

14 congenital diaphragmatic hernias (incidence 1/1597 live births, 12 boys and 2 girls with a sex ratio of 6/1, 10 term infants and 4 preterm first degree, 11 natural births and 3 by caesarean section) admitted to the Clinic of Pediatric Surgery Craiova within a 5-years period (2007-2012), were analyzed from the therapeutic point of view. In the analyzed period, the treatment was based on the recommendations of the diagnosis and treatment guidelines proposed by the "Congenital Diaphragmatic Hernia Study Group" and "CDH EURO Consortium Consensus", including the following stages: prenatal diagnosis, management of the newborn in the labor room, of preoperative respiratory resuscitation and stabilization in the newborn intensive care unit, surgical repair of diaphragmatic defects and postoperative management.

 Prenatal diagnosis was established only accidentally in 4 cases monitored in private practice, the pregnant women being guided for delivery to the university clinics, which were well equipped with logistics and had expertise in the diagnosis and treatment of congenital diaphragmatic hernias. In the rest of the cases, the diagnosis was established immediately after birth, clinically (low Apgar score, respiratory distress, heart and mediastinum displacement, bowel sounds in the chest, etc.) and by imaging tests (plain toraco-abdominal X-ray).

 The management in the labor room started immediately after establishing the diagnosis and severity of respiratory distress and included a set of standard measures (**Table 1**).

**Table 1:** Management in the labor room

Therapeutic measures in the labor room	Cases
IOT	12
Immediately after birth	10
2nd day	1
3rd day	1
Oxygen therapy	14
Naso-gastric tube	14
Vascular access	14
Sedation/analgesia	14

 Oro-tracheal intubation (10 cases with moderate or severe respiratory distress) was followed by mechanical ventilation with a lower peak pressure in the inspired air (< 25 cm H2O), the other 2 cases with moderate respiratory distress were intubated in the second and third day; the administration of oxygen was made under mechanical ventilation (FiO2 = 1.0) in intubated children, never on mask or balloon. The other therapeutic gestures, performed in all cases, placed a naso-gastric suction tube to prevent bowel distension and compression of the lung, safe vascular access and sedation and analgesia set up as soon as venous access was available.

 Preoperative respiratory resuscitation and stabilization (**Table 2**) - set of measures undertaken in the newborn intensive care unit (management of ventilation, pulmonary hypertension and hemodynamic management), aiming to obtain a biological status allowing the performance of the surgical repair with minimal risk for the patient. 

**Table 2:** Preoperative resuscitation and stabilization in newborn intensive care unit

Preoperative resuscitation and stabilization in newborn ICU	Cases
Conventional mechanical ventilation (CMV)	12
SIMV	4
IPPV	8
Pulmonary hypertension management	12
Fluid therapy	12
Transfusion	3
Inotropic drugs	7
Antibiotic	14
Sedation + analgesic	14
Duration	1-5 days (2.8 on average)

 Ventilatory strategy based on the concept of "gentle mechanical ventilation with permissive hypercapnia" aimed at achieving the following parameters: preductal saturation of 85-95%, postductal saturation of 70% and PaCO2 40-60%. Conventional mechanical ventilation (CMV) was used in 12 cases, the only one allowed by our fan performance (Infant Ventilator "Serachrest"), with the following parameters: PIP ≤ 25 cm H2O, PEEP = 2-5 mmHg, FiO2 = 0.36-1 (average 0.42), adjusting the ventilation rate to achieve PaCO2 = 45-60 mmHg. The types of mechanical ventilation were SIMV (Synchronous Intermittent Mandatory Ventilation) in 4 cases and IPPV (Intermittent Positive Ventilation Pressure) in 8 cases. Two infants with mild respiratory dysfunction required no mechanical ventilation and they were placed in an incubator, and a slight increase of the oxygen fraction in the inspired air was sufficient. 

 The treatment of the pulmonary hypertension was set up when the preductal saturation fell below 85% and the signs of ineffective tissue perfusion were present (pH < 7.25, PaCO2 > 80 mmHg) and included fluid therapy (12 cases), transfusion (3 cases) and inotropic drugs (7 cases).

 Hemodynamic management, which was meant to ensure an adequate tissular perfusion (assessed on the following parameters: heart rate according to gestational age, capillary refill less than 3 seconds, the urine output > 1 ml/kg/h and serum lactate <3 mmol/l), sedation and analgesia avoiding muscle blockers and antibiotics, were the measures and therapeutic means which completed the preoperative resuscitation and stabilization. 

 The duration of the preoperative resuscitation and stabilization ranged from 1 to 5 days (2.8 days on average).

 Surgery, the main therapeutic sequence, was aimed at reducing the herniated viscera into the abdomen and closing the diaphragmatic defect, thus allowing the lung expansion and the correction of the pulmonary hypoplasia with the growth and the development of the lung. All the patients were operated on and, although surgical repair of the congenital diaphragmatic hernia is generally a relatively simple problem, several surgical technical and tactical decisions had to be taken depending on the particularities of each case.

**Graph 1:**
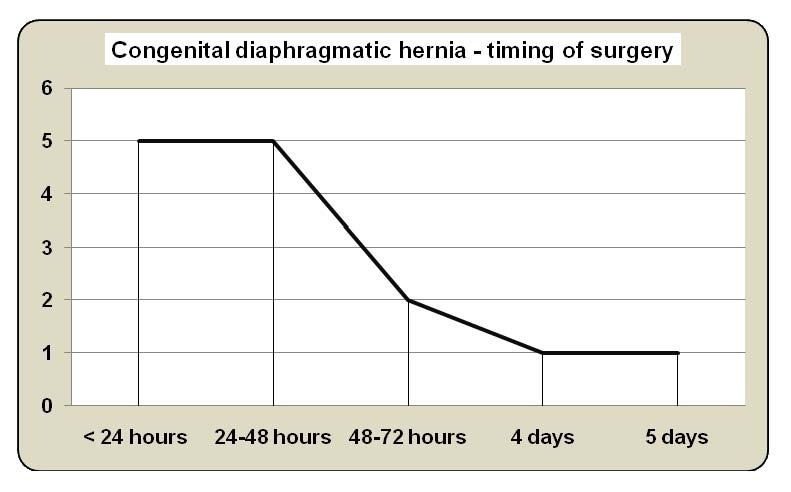
Timing of surgery operator

 - Timing of the surgical repair (**Graph 1** and **Table 3**) - 2.8 days on average, ranged from 12 hours to 5 days, were established by the surgeon together with the anesthesiologist and the newborn expert, corresponding to the achievement of the efficiency parameters of preoperative stabilization therapy (mean arterial pressure normal for gestational age, preductal saturation of 85-95% on FiO2 0.5, serum lactate concentration < 3 mmol/l and urine output > 2 ml/kg/hour).

**Table 3:** Surgical treatment

TIMING OF SURGERY	2.8 days on average
APPROACH	
Median laparotomy	10
Subcostal incision	3
Thoracoscopy	1
TOPOGRAPHY	
Left-sided	12
Right-sided	2
CDH Study Group classification	
Type A	1
Type B	10
Type C	3
HERNIATED VISCERA	
Small bowel	14
Colon	7
Right	2
Transverse	2
fully	3
Stomach	3
Liver	2
Right lobe	1
Left lobe	1
Spleen	10
NUMBER OF HERNIATED VISCERA	
1	3
2	7
3	4
4	1
5	1
Closing of the diaphragmatic defect + Primary tension free suture	14
PLEURAL DRAINAGE	6

**Fig. 1:**
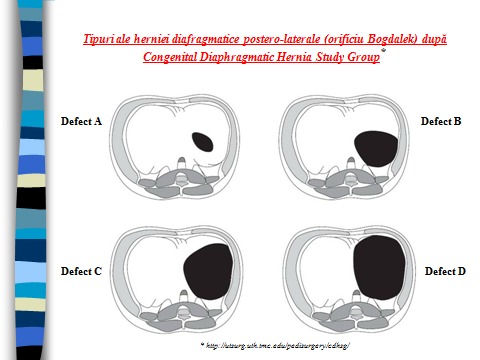
The postero-lateral congenital diaphragmatic hernia (Bogdalek) type - CDH Study Group classification

 - General anesthesia with oro-tracheal intubation in all cases by using inhaled agents (with favorable effect on pulmonary vasculature), opioids and muscle relaxants. 

 - Surgical approach (**Table 3**): xifo-umbilical median laparotomy (10 cases), subcostal incision (3 cases) and thoracoscopy (1 case, converted).

 - The intraoperative exploration allowed us to establish the side of the hernia, the number and type of the herniated viscera (**Table 3**), the evaluation of the size of the defect and quality of its margins, and then its framing in the classification of the postero-lateral congenital diaphragmatic hernia (Bogdalek), proposed by the CDH Study Group (**Fig. 1**), which is useful in choosing the surgical procedure of closing the defect. We registered 12 left-sided and 2 right-sided diaphragmatic hernias; the small intestine was the most common herniated viscus (14 cases), followed by the spleen (10 cases), colon (7 cases), stomach and liver (3 respectively 2 cases), the most common combination being small intestine, spleen and colon. Regarding the type of defect, we registered type A 1 case, type B 10 cases and type C 3 cases.

 - "tension free" primary suture was the procedure used for closing the defect in all cases.

 - pleural drainage Beclaire type (6 cases) was maintained for 6 days on average, until radiologic control showed lung expanding and no fluid or air pleural collections. 

 - Abdominal wall closure was anatomically made in all cases.

 The postoperative care continued the goals and directions, which were set preoperatively, the therapeutic approach being based on the condition of the newborn. The continuation of mechanical ventilation (9 cases) was determined by the severity of the pulmonary dysfunction, quantified by the difference (A-a)DO2. In 6 cases with the difference (A-a)DO2 < 400 mmHg, the conventional mechanical ventilation was continued for a period of 2-12 hrs, while in 3 patients with the difference (A-a)DO2 > 500 mmHg, the mechanical ventilation was continued for a period of 2-10 days. The parameters used were PIP = 18 cm H2O, PEEP = 4 cm H2O, FIO2 = 0.5 and respiratory rate 40. The other measures included fluid therapy, closely monitored and restricted in the first 24 hours to 40 ml/kg/day, including medication, diuretics in cases with positive fluid balance and resuming enteral feeding after restoring the intestinal transit.

## Results 

The results (**Table 4**): 64.29% survival rate, postoperative mortality rate of 35.71% and postoperative morbidity rate of 50% can be considered as acceptable for a center with a low number of operated cases, limited experience and technical equipment, as compared with centers of excellence with high performance equipment (fans performing inhaled nitric oxide and ECMO) and an addressability rate higher than 15 cases/year. 

**Table 4:** Results

Morbidity/Mortality/Survival	Cases	%
POSTOPERATIVE MORBIDITY		
RATE	7	50
Acute respiratory distress (ARDS)	4	
Pneumothorax	1	
Evisceration	2	
POSTOPERATIVE MORTALITY		
RATE	5	35.71
Severe pulmonary hypoplasia + irreversible ARDS	2	
Severe congenital cardiac malformations	1	
Bilateral pneumothorax	1	
Sepsis	1	
SURVIVAL RATE	9	64.29

 Analyzing the structure of morbidity and mortality, it was noticed that pulmonary hypoplasia and secondary pulmonary hypertension were the main postoperative complications and causes of death, together with postoperative pneumothorax and severe cardiac malformations.

## Discussions

The improvement of the outcomes in congenital diaphragmatic hernia was possible only after the understanding of the pathogenesis and pathophysiology of the disease, deeply modified the therapeutic concept and replaced the old concept that considers surgical repair as an immediate emergency with the modern concept of delayed surgery, preceded by the preoperative respiratory resuscitation and stabilization, a therapeutic approach that, in accordance with diagnosis and treatment guidelines proposed by CDH and CDH EURO Consortium Study Group Consensus, set out the next steps of the standard therapeutic algorithm [**1-4**]: 

 - prenatal diagnosis, monitoring pregnancy and birth;

 - management of the newborn in the labor room;

 - preoperative resuscitation and stabilization of the newborn in an intensive care unit;

 - surgical repair;

 - postoperative management.

 Prenatal diagnosis of the congenital diaphragmatic hernia was possible once the ultrasound test was introduced as a main imaging method for the monitoring of the pregnancy; it allowed the evaluation of the evolution predictors of the pregnancy to assess the survival chances of the newborn and to guide the pregnant woman for delivery to a performing center with technical equipment and expertise in neonatology, neonatal intensive care and pediatric surgery. Unfortunately, in Romania, this examination has not become a routine yet, the prenatal diagnosis being established in our group only accidentally in 4 cases monitored in the private praxis [**3-5**].

 The management in the labor room [**3,4,6**] involved the oro-tracheal intubation in the newborn with moderate or severe respiratory distress to diminish the risk of the emphasis of the pulmonary hypertension due to high superimposed acidosis and hypoxia, followed by mechanical ventilation with lower peak pressure in the inspired air (< 25 cm H2O). The oxygen therapy was done during the mechanical ventilation (FiO2=1.0) in intubated children, never using a mask or balloon, that can increase gastric and intestinal distension exacerbating the lung compression. The other therapeutic gestures recommended before the newborn left the delivery room are placing a naso-gastric suction tube to prevent bowel distension and compression of the lungs, thus ensuring a safe vascular access and sedation and analgesia instituted as soon as venous access was available.

 The preoperative resuscitation and stabilization, carried out in the newborn care unit service, precede surgery and include three basic directions: management of ventilation, treatment of the pulmonary hypertension, and hemodynamic management, aiming to achieve a stable biological status that can provide low-risk surgery conditions.

 Ventilatory strategy was based on the concept of "gentle mechanical ventilation with permissive hypercapnia", aiming to achieve the following parameters: pre-ductal saturation of 85-95%, postductal saturation of 70% and PaCO2 40-60% [**3,4,6,8**]. The conventional mechanical ventilation (CMV), the only allowed by our fan performance (Infant Ventilator "Serachrest"), was performed in all our cases, with the following parameters: PIP ≤ 25 cm H2O, PEEP = 2-5 mmHg, FiO2 = 0.36-1 (0.42 on average), adjusting the ventilation rate to achieve PaCO2 = 45-60 mmHg. Because of the performance of our fan shape, we had no experience in the HFOV (High Frequency Oscillatory Ventilation) indicated in cases that required PIP > 28 cm H2O to obtain the above mentioned parameters; although some authors used it as the primary ventilatory strategy, the indications of HFOV are still not clear and it is mainly used as a rescue therapy for persistent hypoxemia and hypercapnia in resistant CMV cases [**3**]. 

 The treatment of the pulmonary hypertension, established when the preductal saturation fell below 85% and the signs of the ineffective tissular perfusion occured (pH < 7.25, the PaCO2 > 80 mmHg), the fluid therapy for ensuring an adequate tissular perfusion, the transfusion for optimizing tissue oxygenation and inotropic drugs (dopamine, dobutamine, and epinephrine) for maintaining the blood pressure at the appropriate gestational age (> 40 mmHg) were available as a conventional therapeutic means. Persistent pulmonary hypertension needed pulmonary vasodilator therapy, inhaled nitric oxide (iNO) being the first option. A potent vasodilator, the nitric oxide had an immediate and spectacular effect, but transient, and, although it was considered the "golden standard" of pulmonary hypertension treatment, 30% of the cases remained without any therapeutic answer and there is evidence showing a difficulty in controlling pulmonary hypertension recurrence after the NO disconnection, so that its administration should be reserved for patients with pulmonary hypertension overcoming the systemic level [**4,9,6,10**]. We had no experience and we did not comment upon the efficiency and opportunity of the other pulmonary vasodilators, such as prostacyclin and prostaglandin E1, used in cases without any therapeutic response at inhaled NO, or such as sildenafil, endothelin antagonists, and tyrosine-kinase inhibitors, used rather in the chronic phase of pulmonary hypertension in congenital diaphragmatic hernia.

 Hemodynamic management designed to ensure an adequate tissue perfusion (assessed on the following parameters: heart rate according to gestational age, capillary refill less than 3 seconds, the urine output > 1 ml/kg/hour and serum lactate level <3 mmol/l), sedation and analgesia, avoiding muscle blockers, and antibiotics were the therapeutic means which completed the preoperative resuscitation and stabilization. 

 The duration of the preoperative resuscitation and stabilization ranged within very wide limits, from a few hours to a few weeks (2.8 days on average in our study), depending on the severity of the hypertension and pulmonary hypoplasia, on the technical endowment of the newborn intensive care unit and the individual response to therapy, rehabilitation and stabilization.

 The place of surgery in the therapeutic algorithm of congenital diaphragmatic has changed radically in the last 25 years. Historically, congenital diaphragmatic hernia was considered an immediate surgical emergency, aiming to reduce the herniated abdominal viscera and to decompress the lung, an attitude charged with a high postoperative mortality. Deepening the knowledge and understanding the pathophysiology, on the one hand, and improving the management of respiratory failure, on the other hand, have changed the therapeutic strategy, leading to the unanimous acceptance of the concept of delayed surgery preceded by a preoperative resuscitation and stabilization period, credited in the literature with a survival rate of 79-82% [**3,6,11,13**]. 

 Although surgical repair of congenital diaphragmatic hernia is generally a relatively simple problem, there are some controversial points: timing of operation, closure of the defect, pleural drainage, closure of the wall and the indications and opportunity of minimally invasive approach. 

 The timing of operation establishment belongs to the surgeon, anesthetist and newborn expert. Taking into account that the stabilization period varies widely, ranging from several days to several weeks (2.8 days on average in our study) and that as long as there are no universal criteria to define preoperative stabilization, it seems reasonable that surgery should be delayed until the parameters that allow the assessment of the efficiency of the preoperative resuscitation and stabilization therapy are reached (mean arterial pressure normal for gestational age, preductal saturation of 85-95% on FiO2 0.5, lactate <3 mmol/l and urine output > 2 ml/kg/hour), the patient can be operated under minimal risk conditions [**7,9,10,14-17**].

 Although ipsilateral subcostal incision is recommended in literature as the main approach, the median laparotomy was the most often used approach in our study (10 out of 14 cases), taking into account its well-known advantages: no muscle, vessels and nerves interruption, which reduced the risk of immediate and late wound complications; the results (evisceration and 1 postoperative eventration) strengthened our conviction that we chose the correct attitude. We did not use thoracotomy as our primary approach and we did not force to perform a combined abdomino-thoracic approach to reduce the herniated viscera or adhesiolisis. Regarding the miniminvasive approach, our experience is limited to one converted thoracoscopy attempt; moreover, there are still hesitations and controversies regarding the indications for minimally invasive surgery in congenital diaphragmatic hernias, justified by the possibility of cardiopulmonary instability that could be activated by hypercarbia and acidosis. Ideal for older children, presented late, the minimally invasive surgery may be charged with a higher rate of intraoperative incidents and accidents and pathophysiologic complications in newborns [**18,23**]. 

 The intraoperative exploration allowed the location of the defect, to assess the size and quality of margins, particularly important in choosing the type of closure, to evaluate the status of the herniated viscera and reduce it into the abdominal cavity. The left-sided diaphragmatic hernia was the most frequent topographic form (12 cases), the small intestine being the most commonly herniated viscera, followed, in order, by the spleen, colon, stomach and liver; in most cases we noticed two or three intrathoracic herniated viscera, the common association being small intestine, stomach, spleen and/or colon. Blunt polythene tubes, gently passed into the thorax through the defect, allowed air into the chest and equalized intra- and extra-thoracic pressure, thus facilitating the reducing of the herniated viscera.

 The surgical procedure of closing the diaphragmatic defect depends on its size, position and on the quality of its edges, assessed by CDH Study Group classification. Basically, there are three ways of closing the defect:

 "tension free" primary closure by suturing the defect’s edges with nonabsorbable threat, reserved for small defects of type A;

 reconstructive techniques that use neighborhood structures as prerenal fascia, coastal structures or different types of flaps, made of the thoracic or abdominal muscles wall, reserved for type B or C defects [**24-26**];

 prosthetic patch (polypropylene mesh), widely accepted because it offers the possibility of tension-free repairs, thereby reducing abdominal pressure at the moment of the abdominal wall closure, reserved for large defects, type C and D [**27**].

 The "tension free" primary closure of the defect was the chosen method used by us for all types of defects (A, B and C); in the type C defects (12 cases) we used a technical trick introduced by Prof. Alexander Pesamosca in "Marie Curie" Surgery Clinic Bucharest, starting from the fact that, generally, there is an anterior edge of the defect, well individualized, of varying sizes, while the other edge can be reflected and hidden from the peritoneum, so that it has to be found in the retroperitoneal tissue after the incision of this peritoneal fold and the diaphragmatic tissue mobilized. By proceeding this way, we succeeded in closing all the defects, even the C type one, so that it was not necessary to use any of the reconstructive plastic methods. We also did not use the closure of the defect by prosthetic patch to avoid the well-known disadvantages of the prostheses: they do not grow and are charged with a high rate of recurrence (up to 50%), which may be asymptomatic but, on the other hand, they can develop severe evolutive complications such as bowel obstruction or severe respiratory failure [**28,29**].

 Pleural drainage is not necessary, considering that the lung should be allowed to expand gradually and not forced by suction, which can attract mediastinum toward the operated side, creating an over-distension of the controlateral lung and negative pressure suction drainage can add to barotrauma and pulmonary hypertension ventilator induced; it should be reserved only for active bleeding and/or uncontrollable air leakage (6 cases in our study) and if it is necessary it should be a Beclaire type, drainage, not an aspirative one.

 The closure of the abdominal wall can sometimes be a problem; in the large hernias, after the reduction of the hernia contents into the abdominal cavity and closure of the diaphragmatic defect, abdominal wall closure may not be possible or may rise the abdominal pressure at a very high level, leading to the abdominal compartment syndrome, which quickly compromises the respiratory function. In these cases, the closure of the abdominal wall may be limited to skin closure, while the other parietal structures are rebuilt a few months later; if skin cannot be closed without tension, temporary closure can be achieved by using prosthetic materials. We did not experience any difficulties in this regard, always managing an anatomic wall closure without or with an acceptable tension.

 The postoperative care had to continue the goals and directions set preoperatively, the therapeutic approach being based on the condition of the newborn. The continuation of the mechanical ventilation was established by the severity of pulmonary dysfunction, quantified by the difference (A-a)DO2, in addition to other therapeutic measures: fluid therapy, closely monitored and restricted in the first 24 hours to 40 ml/kg/day, including medication, diuretics in cases with positive fluid balance and resume enteral feeding after restoring the intestinal transit.

 The survival rate was difficult to be assessed due to large variations representing significant institutional differences regarding the management strategies (ventilatory techniques, inclusion criteria for ECMO, etc.), timing of surgery, surgical procedures and the increasing number of patients [**30,31**]. Moreover, the presence of associated anomalies, especially congenital heart disease remained a significant risk factor for an unfavorable outcome of these children [**32,33**]. However, the survival rate for children with isolated congenital diaphragmatic hernia has improved significantly in recent decades compared to the historical rate of 50%. Today, although it varies widely (25-83%), the overall survival in children whose birth weight is correlated with a good Apgar score is assessed at 64% while a survival rate of up to 80-93% has been achieved with the rules and current treatment strategies [**33,38**].

 Our results (survival rate of 64.29% and postoperative mortality rate of 35.71%) can be considered fairly good in our center with limited addressability, technical equipment and experience, as compared to centers of excellence that have a high performance equipment (fan performance, inhaled nitric oxide and ECMO) and an addressability of more than 15 cases/year.

## Conclusions

Congenital diaphragmatic hernia is one of the most severe malformations, still charged with high mortality caused by its constant association with hypoplasia and pulmonary hypertension and, at the same time, with other severe malformations.

 Delayed surgery preceded by a period of preoperative respiratory resuscitation and stabilization (24-72 hours on average) significantly reduces postoperative mortality and increases the survival rate. 
